# Supplementary material for: Purine Metabolism and Pyrimidine Metabolism Alteration Is a Potential Mechanism of BDE-47-Induced Apoptosis in Marine Rotifer Brachionus plicatilis
Source: Int J Mol Sci. 2023 Aug 12;24(16):12726. doi: 10.3390/ijms241612726 (PMC10454229; doi:10.3390/ijms241612726)
Supplement: Supplementary file 1 [file ijms-24-12726-s001.zip › ijms-2509012-supplementary.pdf]

Supplementary Materials:

Text S1 Detailed parameters and procedures of LC-MS

The chromatographic conditions: Column temperature 25℃; Flow rate 0.3 mL/min; Injection volume 2 μL. Mobile phase composition A: water + 25mM ammonium acetate + 25mM ammonia water, B: acetonitrile. Gradient elution procedure was as follows: 0-1min, 95%B; 1-14min, B changes linearly from 95% to 65%; 14-16min, B changes linearly from 65% to 40%; 16-18min, B remained at 40%; 18-18.1min, B changed linearly from 40% to 95%; 18.1-23min, B remained at 95%. The samples were placed in an automatic sampler at 4℃ during the whole analysis. In order to avoid the influence of instrument detection signal fluctuation, random sequence is used for continuous analysis of samples. QC samples were inserted into the sample queue to monitor and evaluate the stability of the system and the reliability of experimental data.

Mass spectrometry conditions: Ion Source Gas1 (Gas1): 60, Ion Source Gas2 (Gas2): 60, Curtain gas (CUR): 30, source temperature: 600℃, IonSapary Voltage Floating (ISVF): ± 5500 V; TOF MS scan m/z range: 60-1000 Da, product ion scan m/z range:25-1000 Da, TOF MS scan accumulation time 0.20 s/spectra, product ion scan accumulation time 0.05 s/spectra. Secondary mass spectrometry was obtained by information dependent acquisition (IDA) and used high sensitivity mode. Declustering potential (DP): ± 60 V, Collision Energy: 35±15 eV. IDA set as follows: Exclude isotopes within 4 Da, Candidate ions to monitor per cycle: 6.
